# Association of the Systemic Inflammatory Burden Score With Early and Delayed Dental Implant Failures: A Retrospective Cohort Study

**DOI:** 10.7759/cureus.109587

**Published:** 2026-05-25

**Authors:** Barun Kumar, Tejal Patil, Subachander Prabhakaran, Jasuma Rai, Afrah Fatima, Divya Kadali

**Affiliations:** 1 Department of Oral and Maxillofacial Surgery, Bharati Vidyapeeth (Deemed to be University) Dental College and Hospital, Navi Mumbai, IND; 2 Department of Prosthodontics and Crown and Bridge, Meenakshi Ammal Dental College and Hospital, Chennai, IND; 3 Department of Periodontology, K.M. Shah Dental College and Hospital, Sumandeep Vidyapeeth (Deemed to be University), Vadodara, IND; 4 Department of Orthodontics, Panineeya Institute of Dental Sciences and Research Centre, Hyderabad, IND; 5 Department of Periodontics, Care Dental College and Hospital, Guntur, IND

**Keywords:** dental implants, implant failure, peri-implantitis, risk assessment, systemic inflammation

## Abstract

Introduction: Dental implant therapy is a predictable treatment option; however, implant failures continue to occur owing to a combination of local and systemic factors. The role of cumulative systemic inflammatory burden in influencing implant outcomes has gained increasing attention. This study aimed to evaluate the association between systemic inflammatory burden score (SIBS) and early and delayed dental implant failure.

Materials and methods: This retrospective observational cohort study was conducted using a prospectively maintained institutional database of patients who underwent dental implant placement between January 2005 and December 2023. Adult patients with complete records and a minimum follow-up period of six months were included. Implant failure was categorized as either early (≤12 months) or delayed (>12 months). SIBS was calculated based on diabetes, smoking, periodontitis, and immunosuppression, and it was categorized into low, moderate, and high tiers. Statistical analyses included comparative tests and multinomial logistic regression.

Results: A total of 730 implants were analyzed, of which 635 (86.99%) were successful, 40 (5.48%) showed early failure, and 55 (7.53%) demonstrated delayed failure. Low SIBS (0-3) was predominant in successful implants, whereas high SIBS (8-12) was more frequent in the early and delayed failure groups than in the success group (p < 0.001). High SIBS scores were associated with increased odds of early and delayed failures. Each unit increase in SIBS score increased the odds of overall failure by 18%. Implant diameter was protective against early failure, whereas greater implant length was associated with delayed failure.

Conclusion: The systemic inflammatory burden significantly influenced implant failure, particularly delayed failure. Incorporating SIBS into clinical assessments may improve the risk stratification and treatment outcomes.

## Introduction

Dental implants have become a predictable and widely accepted modality for the replacement of missing teeth, with high long-term survival rates. However, implant failure remains a clinically significant complication, broadly categorized into early failures, which occur during the osseointegration phase, and delayed failures, which arise after functional loading. While local factors such as bone quality, implant design, and surgical technique are well recognized, increasing attention has been directed toward systemic host-related factors that may influence peri-implant healing and long-term stability [1,2]. Chronic low-grade systemic inflammation, often associated with conditions such as diabetes mellitus, smoking, periodontitis, and immunosuppression, may adversely affect bone metabolism, vascularity, and immune response, thereby compromising implant success [3,4].

In this context, the concept of cumulative inflammatory burden has gained importance, as individual risk factors rarely act in isolation. The systemic inflammatory burden score (SIBS) represents a composite measure that integrates multiple systemic conditions into a single quantitative index, potentially offering improved predictive capability compared to isolated variables. Despite this, there is limited evidence evaluating the role of such composite indices in distinguishing between early and delayed implant failures [5,6]. 

Therefore, the present study aimed to evaluate the association between SIBS and dental implant failure, with specific emphasis on differentiating its impact on early versus delayed failures. The objectives of this study were to: (i) assess the distribution of SIBS among successful implants, early failures, and delayed failures; (ii) determine the strength of the association between SIBS tiers and implant failure using multivariable analysis; (iii) evaluate the predictive value of SIBS as a continuous and categorical variable; and (iv) analyze the influence of additional patient-related and implant-related factors on implant outcomes. This study aimed to enhance risk stratification and contribute to personalized treatment planning in implant dentistry.

## Materials and methods

Study design, setting, and ethical approval

This retrospective observational cohort study was conducted using a maintained institutional digital dental archive at the Department of Oral and Maxillofacial Surgery, Bharati Vidyapeeth (Deemed to be University) Dental College and Hospital, Navi Mumbai, India. The study design complied with the Strengthening the Reporting of Observational Studies in Epidemiology (STROBE) guidelines [7]. The dataset included the records of patients who underwent endosseous dental implant placement between January 2005 and December 2023, thereby ensuring a long-term observation window for implant outcomes. Ethical approval was obtained from the Institutional Ethical Committee (BVDUDC&H-IEC-810/2025) prior to data retrieval, and a waiver of informed consent was granted because of the retrospective nature of the study and the use of anonymized patient records in accordance with institutional policies.

Study population

The study population comprised adult patients (≥18 years) who received at least one root-form endosseous titanium dental implant and had a minimum follow-up of six months post-placement or documented implant failure within this period. Only the records with complete baseline clinical, radiographic, and systemic health data were included. Radiographic assessment was performed using standardized panoramic radiographs, intraoral periapical radiographs obtained via the long-cone paralleling technique, and cone-beam computed tomography (CBCT) scans. Records were excluded if implants were placed in irradiated bone or in post-oncologic reconstruction cases, in patients with craniofacial syndromes or primary bone pathology, if implant system details were missing, or if implants were used solely for orthodontic anchorage. Duplicate entries were identified and removed by using a probabilistic matching approach to ensure data integrity.

Outcome assessment

The primary outcome was implant failure, categorized as early implant failure (occurring within 12 months of placement, including failure during osseointegration or early loading) or delayed implant failure (occurring after 12 months of placement). Failure was defined clinically as implant mobility on percussion or reverse torque testing, requirement for implant removal, or radiographic evidence of circumferential bone loss exceeding 50% of the implant length [1,8]. Patient-related variables included age, sex, diabetes status, smoking history, periodontitis severity, and immunosuppression status. Implant-related variables included implant length, diameter, bone quality, anatomical location (maxilla/mandible), augmentation procedures, and the loading protocol. Additional factors, such as prosthesis type, opposing dentition, parafunctional habits, peri-implantitis, and crestal bone loss, were also recorded.

The primary exposure variable was SIBS, a composite index integrating diabetes control (based on glycated hemoglobin (HbA1c) levels), smoking status, periodontitis grade, and immunosuppression status. The conceptual framework for this scoring system was adapted from previously published inflammatory burden indices [5]; however, modifications were made by the authors to suit the clinical context of dental implant outcomes. Each component was scored from 0 to 3, resulting in a cumulative score ranging from 0 to 12. For the analysis, patients were stratified into three categories: low (0-3), moderate (4-7), and high (8-12) inflammatory burden (Table 1). In addition, SIBS was also analyzed as a continuous variable to evaluate dose-response relationships. SIBS is a pragmatic composite reflecting cumulative burden; weighting was kept uniform to avoid overfitting and due to the lack of prior validated weighting models.

**Table 1:** Components and scoring of systemic inflammatory burden score (SIBS) SIBS ranges from 0 to 12. For primary analyses, patients were categorized into three SIBS tiers: low (0-3), moderate (4-7), and high (8-12). DM: diabetes mellitus; HbA1c: glycated hemoglobin

Component	Score 0	Score 1	Score 2	Score 3
Diabetes mellitus (HbA1c)	No DM	DM with HbA1c <7%	DM with HbA1c 7-9%	DM with HbA1c >9%
Tobacco smoking	Non-smoker	Ex-smoker	Current <10 pack-years	Current ≥10 pack-years
Periodontitis grade	No periodontitis	Grade I-II	Grade III	Grade IV
Immunosuppression	None	Systemic corticosteroids	Disease-modifying agents	Transplant-level

Data extraction was performed by trained investigators using a standardized protocol with predefined criteria for all variables. In cases where patients had multiple implants, only one implant per patient was included in the analysis to ensure independence of observations; the selection was based on predefined criteria (such as first placed or randomly selected implant). Any discrepancies during data extraction were resolved through consensus review. Radiographic assessment was conducted using standardized institutional protocols with available imaging modalities (panoramic radiographs, intraoral periapical radiographs, and CBCT where indicated) and predefined criteria for implant failure. The SIBS was constructed as a pragmatic composite index with equal weighting of its components to reflect cumulative inflammatory burden, given the absence of a validated weighting system for implant-specific outcomes.

Sample size estimation

Sample size estimation was performed using G*Power (Heinrich Heine University, Düsseldorf, Germany) for logistic regression analysis, assuming a two-tailed alpha of 0.05, statistical power of 80%, anticipated odds ratio of 1.5, and implant failure prevalence of approximately 9.5%. The minimum sample size required was estimated to be 650 implants. Furthermore, the events-per-variable (EPV) method was applied to ensure model stability, requiring at least nine events per predictor variable. Based on these criteria, a minimum of 711 implants was deemed necessary. The final sample comprised 730 implants, including 71 failure events, satisfying both statistical and modeling requirements.

Statistical analysis

All statistical analyses were performed using IBM SPSS Statistics software (IBM Corp., Armonk, USA). Receiver operating characteristic (ROC) curve analysis was performed using R (R Foundation for Statistical Computing, Vienna, Austria) and Python (Python Software Foundation, Fredericksburg, USA). The normality of continuous variables was assessed using the Shapiro-Wilk test. Normally distributed data were expressed as mean ± standard deviation, whereas non-normally distributed data were presented as medians with interquartile ranges. Categorical variables were summarized as frequencies and percentages. Intergroup comparisons among success, early failure, and delayed failure groups were performed using the Kruskal-Wallis test for continuous variables and the chi-square or Fisher’s exact test for categorical variables.

To evaluate the independent association between SIBS and implant failure, a multinomial logistic regression model was constructed, with implant success as the reference category, adjusting for potential confounders and intra-patient clustering. The SIBS was used as a categorical and continuous variable. Multicollinearity was assessed using the variance inflation factor (VIF), with a threshold of <5 considered acceptable. Model calibration was evaluated using the Hosmer-Lemeshow goodness-of-fit test, while model discrimination was assessed using the area under the ROC curve. A two-tailed p-value of less than 0.05 was considered statistically significant. Sensitivity analyses were performed to validate the robustness of the findings using continuous SIBS values and alternative stratifications.

## Results

A total of 730 dental implants were included in the final analysis, with a mean follow-up duration that differed significantly across the outcome groups. Overall, implant success was observed in the majority of cases, whereas a smaller proportion demonstrated early and delayed failures. The distributions of the baseline demographic and clinical characteristics across the three outcome groups are presented in Table 2. No statistically significant differences were observed with respect to age, sex, or parafunctional habits between the groups. However, implant-related parameters, specifically implant length and diameter, demonstrated significant variations across outcomes, indicating a potential influence on failure patterns. Other surgical and prosthetic variables, including insertion torque, maxillary placement, bone augmentation, bone quality, and loading protocols, did not differ significantly between groups.

**Table 2:** Baseline characteristics of the study cohort according to implant outcome (N = 730 implants). Data presented as mean ± standard deviation (SD), median (IQR), or n (%). H = Kruskal-Wallis test; χ² = Chi-square test. *Statistically significant (p < 0.05). IQR: interquartile range

Variable	Total (N = 730)	Early failure (n = 40)	Late failure (n = 55)	Success (n = 635)	Test value	p-value
Patient characteristics						
Age (years), mean ± SD	48.9 ± 12.0	46.8 ± 11.8	49.0 ± 11.8	49.0 ± 12.0	H = 0.45	0.797
Male sex, n (%)	397 (54.4%)	15 (62.5%)	24 (51.1%)	358 (54.3%)	χ² = 0.85	0.655
Follow-up (months), median (IQR)	63.0 (39.4-92.3)	6.1 (4.1-8.9)	52.4 (31.5-69.4)	66.7 (41.8-94.9)	H = 112.6	<0.001*
Parafunctional habits	183 (25.1%)	5 (20.8%)	8 (17.0%)	170 (25.8%)	χ² = 2.03	0.361
Implant characteristics						
Length (mm), mean ± SD	11.1 ± 2.0	10.6 ± 1.9	11.8 ± 1.9	11.0 ± 2.0	H = 7.72	0.021*
Diameter (mm), mean ± SD	4.1 ± 0.6	3.8 ± 0.6	4.2 ± 0.5	4.1 ± 0.6	H = 10.46	0.005*
Insertion torque (Ncm), mean ± SD	32.3 ± 8.8	32.1 ± 9.2	33.9 ± 8.0	32.2 ± 8.8	H = 1.45	0.485
Surgical/prosthetic variables						
Maxillary placement, n (%)	326 (44.7%)	9 (37.5%)	24 (51.1%)	293 (44.5%)	χ² = 1.29	0.525
Immediate loading, n (%)	115 (15.8%)	3 (12.5%)	11 (23.4%)	101 (15.3%)	χ² = 3.09	0.213
Bone augmentation (any), n (%)	310 (42.5%)	10 (41.7%)	22 (46.8%)	278 (42.2%)	χ² = 0.39	0.823
Bone quality III/IV, n (%)	384 (52.6%)	12 (50.0%)	29 (61.7%)	343 (52.0%)	χ² = 1.71	0.426

The distribution and comparative analysis of the SIBS scores across implant outcomes are summarized in Table 3. A statistically significant association was observed between an increasing SIBS score and implant failure (p < 0.001). The proportion of individuals with a higher inflammatory burden was notably greater in both the early and delayed failure groups than in the successful implants group, demonstrating a clear gradient effect. Post-hoc analysis confirmed that both the early and delayed failure groups differed significantly from the success group, whereas differences between early and delayed failures were limited for most SIBS components. 

**Table 3:** Distribution of systemic inflammatory burden score (SIBS) according to implant outcome. H = Kruskal-Wallis test; χ² = Chi-square test. *All p‑values remained significant after Benjamini‑Hochberg false discovery rate (FDR) correction for eight tests (critical q = 0.05).

Variable	SIBS component/score	Success (n = 635)	Early failure (n = 40)	Late failure (n = 55)	Test statistic	p‑value
SIBS (mean ± SD)	Total score	2.9 ± 2.2	6.4 ± 2.5	7.2 ± 2.8	H = 116.47	<0.001*
SIBS tier, n (%)	Low (0-3)	395 (62.2%)	8 (20.0%)	7 (12.7%)	χ² = 112.85	<0.001*
	Moderate (4-7)	195 (30.7%)	18 (45.0%)	27 (49.1%)		
	High (8-12)	45 (7.1%)	14 (35.0%)	21 (38.2%)		

Multinomial logistic regression analysis evaluating the independent effects of SIBS and other covariates on implant failure is presented in Table 4. After adjustment for confounding variables, a high SIBS score was significantly associated with increased odds of both early and delayed implant failure, with a stronger association observed for delayed failure. Moderate SIBS was significantly associated with delayed failure but not with early failure. Among implant-related variables, a greater implant diameter was found to be protective against early failure, whereas increased implant length was associated with a higher likelihood of delayed failure. No statistically significant associations were identified between age, sex, bone quality, implant location, loading protocol, augmentation procedures, or parafunctional habits.

**Table 4:** Multinomial logistic regression analysis for implant failure. Model fit statistics: -2 log likelihood = 468.2; Nagelkerke R² = 0.19; overall model χ² = 42.6, p < 0.001. No multicollinearity detected (all VIF < 2.5). Reference category: SIBS low (0-3). *Statistically significant (p < 0.05). VIF: variance inflation factor; OR: odds ratio; CI: confidence interval; SIBS: systemic inflammatory burden score

Variable	Early failure vs. success			Late failure vs. success		
	Adjusted OR (95% CI)	Wald χ²	p-value	Adjusted OR (95% CI)	Wald χ²	p-value
SIBS tier Low (0-3)	Reference	–		Reference	–	
Moderate (4-7)	1.85 (0.78-4.38)	1.98	0.16*	2.40 (1.10-5.20)	4.84	0.02*
High (8-12)	3.20 (1.05-9.75)	4.18	0.04*	4.85 (2.05-11.50)	12.60	0.001*
Age (per 10 years)	1.10 (0.75-1.62)	0.24	0.62	1.18 (0.88-1.58)	1.21	0.27
Male sex	1.28 (0.55-2.95)	0.35	0.56	0.95 (0.48-1.85)	0.03	0.87
Implant length (per 1 mm)	0.88 (0.72-1.07)	1.71	0.19	1.18 (1.03-1.35)	5.64	0.02*
Implant diameter (per 1 mm)	0.64 (0.41-0.99)	3.94	0.04*	1.22 (0.82-1.82)	0.96	0.31
Bone quality (D3/D4 vs. D1/D2)	0.92 (0.40-2.11)	0.04	0.84	1.45 (0.78-2.68)	1.45	0.23
Maxilla (vs. mandible)	0.78 (0.33-1.85)	0.30	0.58	1.25 (0.68-2.30)	0.52	0.47
Augmentation procedure	0.95 (0.39-2.33)	0.01	0.91	1.20 (0.63-2.29)	0.30	0.58
Immediate loading (vs. conventional)	0.82 (0.23-2.85)	0.10	0.75	1.75 (0.85-3.60)	2.28	0.13
Parafunctional habits	0.72 (0.26-1.95)	0.42	0.52	0.65 (0.30-1.40)	1.22	0.27

Sensitivity analysis of SIBS as a continuous variable (Table 5) demonstrated a consistent dose-response relationship, wherein each unit increase in SIBS was associated with a statistically significant increase in the odds of overall implant failure and delayed failure. Although a similar trend was observed for early failure, it did not reach statistical significance after the adjustment.

**Table 5:** Sensitivity analysis of systemic inflammatory burden score (SIBS) as a continuous variable. Adjusted for age, sex, implant length, bone quality, and loading protocol. *Statistically significant (p < 0.05). OR: odds ratio; CI: confidence interval

Outcome	Unadjusted OR (95% CI)	Wald χ²	p-value	Adjusted OR (95% CI)	Wald χ²	p-value
Any failure vs. success	1 1.22 (1.10-1.35)	102.45	<0.001	1.18 (1.06-1.31)	48.22	0.002*
Early failure vs. success	1.18 (1.02-1.36)	52.33	0.022	1.14 (0.98-1.33)	22.89	0.085
Late failure vs. success	1.25 (1.12-1.40)	81.67	<0.001	1.21 (1.08-1.36)	38.45	0.001*

**Figure 1:**
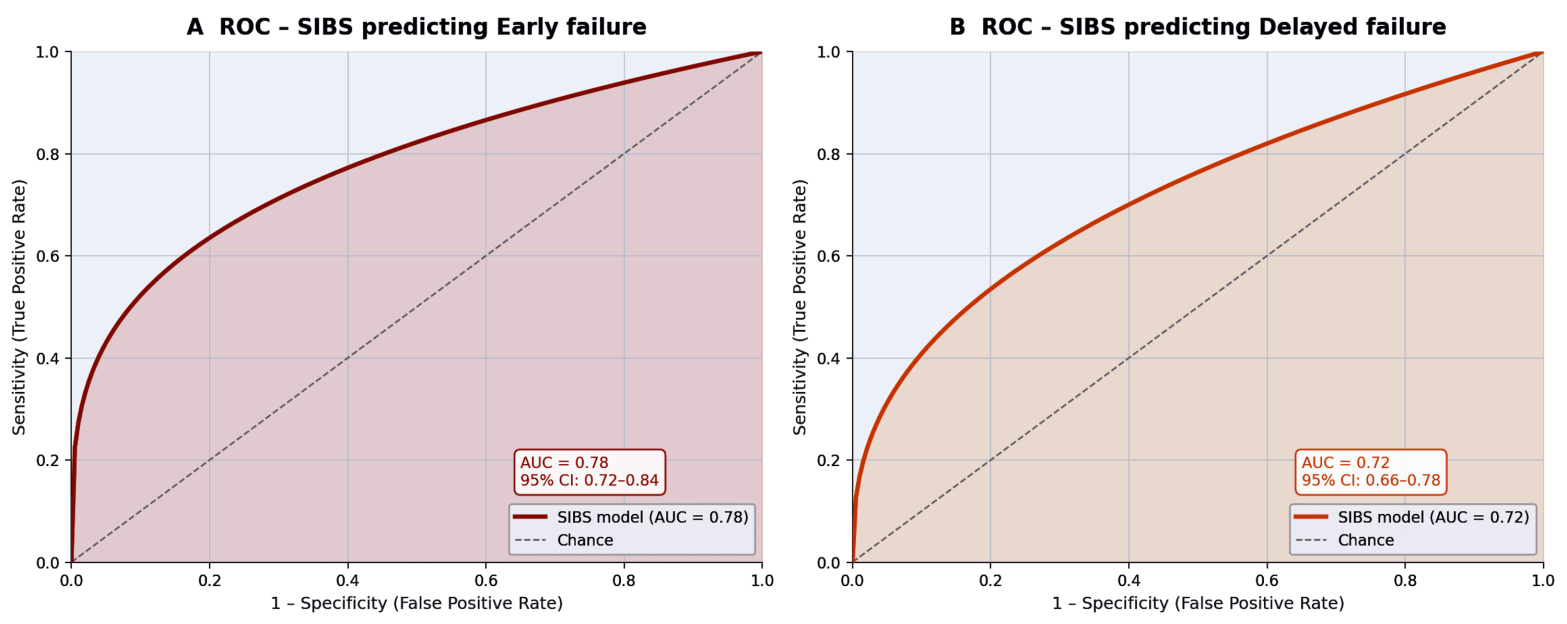
Receiver operating characteristic (ROC) curves for early implant failure (A) and delayed implant failure (B). AUC: area under the curve; SIBS: systemic inflammatory burden score; CI: confidence interval

The discriminative ability of SIBS to predict implant failure is illustrated in Figure 1 (ROC curves). The SIBS demonstrated good predictive performance for early implant failure and acceptable discrimination for delayed failure, supporting its potential utility as a clinically relevant risk stratification tool.

## Discussion

The present study evaluated the association between SIBS and the risk of early and delayed dental implant failure, and demonstrated a strong and graded relationship between increasing systemic inflammatory burden and implant failure. These findings indicate that SIBS is not only associated with implant failure but also exhibits a differential impact on early versus delayed failure, with a more pronounced effect observed in delayed implant loss.

The observed association between a higher SIBS and implant failure can be explained by the cumulative effect of systemic inflammatory conditions on bone metabolism and wound healing. Chronic systemic inflammation, as observed in poorly controlled diabetes, smoking, and periodontitis, is known to impair osteoblast function, enhance osteoclast activity, and disrupt angiogenesis [3,9-11]. This imbalance adversely affects osseointegration, particularly during the early healing phase, thereby increasing the susceptibility to early implant failure. At the same time, a persistent inflammatory burden may contribute to progressive peri-implant bone loss and peri-implantitis, explaining its stronger association with delayed failures.

Interestingly, the present study demonstrated that, while both moderate and high SIBS were associated with delayed implant failure, only high SIBS showed a statistically significant association with early failure. This suggests that a threshold level of systemic inflammation may be required to disrupt initial osseointegration, whereas moderate inflammation may be sufficient to influence long-term peri-implant tissue stability. This finding aligns with the concept that early implant failure is more dependent on immediate healing conditions, whereas delayed failure reflects chronic biological and biomechanical influences over time [12].

The dose-response relationship observed in the sensitivity analysis further strengthens the validity of the SIBS as a continuous risk indicator. Each incremental increase in SIBS score was associated with a proportional increase in implant failure risk, supporting the hypothesis that systemic risk factors exert an additive rather than an isolated effect. This is consistent with previous studies that have highlighted the synergistic impact of diabetes and smoking on implant outcomes as well as the role of periodontitis as a risk modifier for peri-implant disease [13,14].

From an implant-related perspective, the finding that increased implant diameter was protective against early failure may be attributed to improved primary stability and a greater bone-implant contact area, which are critical during the osseointegration phase [15]. Conversely, the association between increased implant length and delayed failure may reflect confounding factors such as placement in compromised bone conditions or increased biomechanical loading in longer implants [16]. These findings underscore the multifactorial nature of implant success and the need to interpret implant dimensions in conjunction with systemic and local factors. The lack of a significant association between variables such as age, sex, bone quality, and loading protocol with implant failure in this study may be due to adequate case selection and standardized clinical protocols within the institution. It is also possible that the effect of systemic inflammatory burden overshadows these factors, highlighting its relative importance as a determinant of implant outcomes.

The predictive performance of SIBS, as demonstrated by ROC analysis, indicates that it has a good discriminative ability for early failure and acceptable performance for delayed failure. This suggests that SIBS could serve as a clinically useful and easily applicable tool for preoperative risk assessment [17]. Unlike single risk factors, the SIBS integrates multiple systemic conditions into a single composite score, providing a more holistic evaluation of patient risk. This aligns with contemporary trends in personalized and precision medicine, in which cumulative risk profiling is increasingly emphasized.

The findings of this study have important implications. Preoperative assessment of the systemic inflammatory burden could aid clinicians in identifying high-risk patients and implementing tailored treatment strategies, such as optimizing glycemic control, encouraging smoking cessation, and managing periodontal disease prior to implant placement. Additionally, patients with high SIBS scores may benefit from modified treatment protocols, including delayed loading, enhanced maintenance programs, and closer follow-up to mitigate the risk of failure. The use of SIBS may also improve patient counseling by providing a quantifiable estimate of risk.

This study has several limitations. Its retrospective, single-center design introduces potential selection bias and limits generalizability. Although one implant per patient was included to ensure independence of observations, residual confounding from unmeasured variables such as oral hygiene practices, maintenance compliance, implant system/surface characteristics, surgeon experience, prosthetic design, and history of peri-implantitis cannot be excluded. The SIBS is an author-constructed composite index with equal weighting of components and has not been externally validated for implant outcomes; therefore, its findings should be considered exploratory. Additionally, the minimum follow-up period of six months may limit the complete capture of late implant failures, and variations in clinical protocols over the extended study period may have influenced outcomes.

## Conclusions

Within the limitations of this retrospective cohort study, the SIBS demonstrated a significant and independent association with dental implant failure. Higher SIBS values were strongly linked to increased risk, particularly for delayed implant failure, with a clear dose-response relationship observed. While early failures require a higher inflammatory threshold, even a moderate systemic burden influences the long-term outcomes. These findings highlight the importance of cumulative systemic health assessments in implant dentistry. Incorporating SIBS into preoperative evaluations may enhance risk stratification, enable personalized treatment planning, and improve long-term implant success through targeted preventive and maintenance strategies.
